# A Review of Guidelines to Resuming Elective Orthopaedic Surgeries Amid COVID-19 Pandemic: Deriving a Simple Traffic Light Model

**DOI:** 10.5704/MOJ.2011.003

**Published:** 2020-11

**Authors:** AK Attia, UF Omar, AK Kaliya-Perumal

**Affiliations:** 1 Department of Orthopaedic Surgery, Hamad Medical Corporation, Doha, Qatar; 2 Department of Orthopaedic Surgery, Khoo Teck Puat Hospital, Singapore; 3 Lee Kong Chian School of Medicine, Nanyang Technological University Singapore, Singapore

**Keywords:** coronavirus, COVID, infectious disease, orthopaedic surgery, pandemic

## Abstract

The COVID-19 pandemic has affected most healthcare systems around the world. Routine care operations such as outpatient clinics and elective surgery remain badly hit. This situation cannot continue for long as it puts patients at a higher risk for complications due to delayed management. Hence, it is essential to resume routine, especially elective surgery. Regarding orthopaedic practice, various authors and organisations have come out with guidelines to resume elective surgeries. However, clear consensus and common strategies need be derived. With this motive, we conducted a review of the literature for guidelines to resume elective orthopaedic surgery amid COVID-19 pandemic and shortlisted scholarly publications and information from regional organisations. We have summarised the information and derived an organised algorithm considering the guidelines provided by various sources. In this extraordinary time, guidelines come in as a relief for every surgeon who is in a dilemma whether to resume electives or not. Putting safety first, these guidelines or suitable versions should be followed at all levels wherever possible to avoid the lack of trained manpower in the event of staff morbidity.

## Introduction

Coronavirus Disease 2019 (COVID-19), referred to as "the modern pandemic", has so far been catastrophic on a level that we have not witnessed for many years^1^. Is has disrupted health, economy, and routine to a great extent^2^. The healthcare profession was not spared, and routine care operations were badly hit throughout the world. While surgical specialties at most institutions continued to perform emergency surgeries, elective procedures were temporarily stopped so as to reduce hospital visits by patients^3^. This also facilitated the health care manpower, including doctors, nurses, and paramedical staff, to be redirected to COVID-19 tasks owing to the overwhelming case load^4^.

Orthopaedic surgeons around the world have followed this general rule to reduce in-hospital COVID-19 transmission risk and restricted themselves to procedures that are deemed an emergency. Worldwide, an estimate of six million orthopaedic surgeries has been cancelled or postponed due to COVID-19^5^. Operated were only the conditions that risk a complication if left untreated, such as open/major fractures, tumours, medically uncontrollable infections, and acute conditions compromising vascularity or neurology^6^. Now that many nations are entering the post-peak phase of the pandemic, it is necessary to investigate ways to resume elective surgery because certain conditions tend to worsen if not intervened timely. However, resuming electives will be a walk on a tightrope as there is always a risk for disease transmission in a hospital setup until COVID-19 is eradicated. Hence, a guideline is paramount to resuming elective surgeries safely.

Orthopaedic surgeons and related staff need to be well informed of the procedures that can be performed, steps to be taken before, during, and after the procedure, risks involved, and personal protective measures to cope with the long waiting lists. While various authors and organisations have proposed guidelines based on the current situation, it is necessary that a universal standard is derived^7-11^. With this motive, we have shortlisted key information from the available literature and intend to provide a dashboard of common strategies that can be adopted by orthopaedic surgeons worldwide to resume electives once the pandemic palliates.

## Literature Review

### Search strategy

Relevant studies in English literature were identified from database inceptions until May 2020 for which an electronic-based search was conducted on MEDLINE (PubMed), EMBASE, Google Scholar, and Cochrane databases using the following keywords with their synonyms: ("resuming elective surgery" AND "COVID-19" AND "guidelines" AND "orthopaedic surgery"). In addition, the reference lists from previous articles were searched manually to check for eligible studies. Furthermore, orthopaedic societies' websites were manually searched for eligible guidelines. These websites included the American Academy of Orthopaedic Surgeons (AAOS), Royal College of Surgeons (RCS), European Federation of National Associations of Orthopaedics and Traumatology (EFORT), American Orthopaedic Foot and Ankle Society (AOFAS), American Orthopaedic Society for Sports Medicine (AOSSM), International Society of Arthroscopy, Knee Surgery and Orthopaedic Sports medicine (ISAKOS), Société Internationale de Chirurgie Orthopédique et de Traumatologie (SICOT).

### Inclusion and exclusion

Investigators independently reviewed the information on 1) The impact of COVID-19 on orthopaedic practice, 2) Recommendations for performing elective orthopaedic surgeries and 3) The role of telemedicine for pre and postoperative care from scholarly publications and international orthopaedic societies websites. Based on abstract review, full texts were shortlisted and studied in detail. Studies not reporting any of the variables of interest or if the full text is not available in English were excluded. Any disagreement was resolved by discussion, and all decisions were unanimous.

## Guidelines for Resuming Elective Orthopaedic Surgeries

Based on the recommendations provided by various sources, the following key areas were identified, and corresponding necessities under each key area is mentioned.

### Infrastructure

Infrastructure is considered the most important as we prepare strategically for resuming elective operations. Without proper infrastructure, other key measures may fail.

Hence, it is necessary to build up an appropriate infrastructure with the following amenities^12,13^,

1. Establishing of COVID-19 negative pathway for elective cases (Separate building or unit that is not in proximity to a COVID-19 ward and has restricted entry to those performing COVID-19 tasks).
2. The availability of isolation areas, recovery rooms, and negative pressure theatres for operating on COVID-19 positive patients.
3. The availability of adequate ICU beds, preferably as separate rooms.
4. The hospital preparedness status (availability of a strategic stockpile) to deal with a potential 2nd wave of COVID-19.
5. Strictly administered physical distancing measures inside hospital premises, including clinics, operative theatre waiting areas, and recovery rooms.

### Healthcare workforce

Reported evidence suggests that in most hospitals, which were COVID-19 centers, a majority of the manpower was redirected to COVID-19 tasks during the pandemic. As they return to routine practice, they should be COVID free and protected.

Hence to facilitate this,

1. Returning staff should be tested before resuming electives and periodically on a weekly basis, either using SARS-CoV real-time reverse transcription polymerase chain reaction (rRT-PCR) test or rapid kit tests to check for antibodies depending on availability.
2. It is necessary to make sure that the entire caregiving team, including the surgeon, anesthetist, nurses, physiotherapists, are available and disease-free prior to performing an elective procedure.

### Resources

Mobilising the necessary resources can be an economic burden but of significant importance. Hospitals should invest in arranging these to prevent disease transmission inside the hospital set up, especially during procedures^14-16^.

The most important resources include,

1. Personal protective equipment (PPE)
2. Additional surgical instruments and supplies.
3. Equipment for infection control.
4. COVID-19 testing ability of the hospital in addition to other diagnostic services.

### Patient selection and observation

Once the above key areas are strengthened, patients can be shortlisted for elective procedures. If everything is favorable, firstly, patients with deteriorating symptoms or those with a high risk of a complication if delayed further should be selected. Secondly, previously cancelled or postponed patients due to COVID-19 situation can be prioritised rather than listing new patients. However, even though if the situation may seem favorable, reservations should be kept for if a second wave has to be encountered. Hence, beds should always be available. This puts hospitals in a difficult situation to select the most deserving patients for surgery and delay the rest. In addition, selected patients need to be monitored on a remote basis.

Guidelines suggest to^1,17-19^,

1. Avoid asymptomatic COVID-19 positive patients if infrastructure, manpower, or resources do not favor operating on such patients.
2. Obtain detailed history on travel, occupation and contact history
3. Test every patient before scheduling or within one week prior to admission, whichever is later, preferably with SARS-CoV real-time reverse transcription polymerase chain reaction (rRT-PCR) test as rapid kit tests to check the antibodies are not recommended for use in the surgical patients due to their variable results.
4. Select patients depending on COVID-19 exposure history and testing result in addition to age and American Society of Anesthesiologists (ASA) classification.
5. Obtain appropriate informed consent, clearly explaining the additional risk of infection.
6. Monitor patients’ temperature on a daily basis and watch out for COVID-19 symptoms until the day of surgery.
7. Minimise patient visits to the hospital by using teleconsultation wherever possible.
8. Provide patients with information about COVID-19 symptoms and ways of transmission.
9. Instruct strictly about the importance of using face mask and hand hygiene.

### Intra-operative strategies

Operative preparations may vary depending on the patient's COVID-19 test status. While additional PPE may be needed when operating on a COVID-19 positive patient, certain universal precautions need to be taken irrespective of patient status. These measures are of extreme importance to protect healthcare professionals from being exposed to COVID-19, which will have a severe impact on the healthcare manpower of the hospital.

Hence, sources suggest that^13,20-22^,

1. COVID-19 positive and suspected patients are to be operated only in dedicated operating theatres that are negative pressure rooms (NPRs).
2. These NPRs shall not be allotted for procedures on non-COVID-19 patients, while they can be managed in regular operating rooms with additional precautions.
3. Local and regional anaesthesia are to be preferred over general anesthesia (GA), which requires intubation.
4. If GA is performed, the number of people inside the theatre, especially during intubation and extubation, should be minimised.
5. Surgeons should consider the judicial use of electric cautery and suction to minimise aerosolization.
6. Surgeons should minimise equipment use and open only the packs that are needed.
7. Skin closure can be done with absorbable sutures, so that post-operative follow-up visits are minimised.
8. Operating rooms, irrespective of patient's COVID-19 status, should be thoroughly disinfected after each procedure.

It should also be noted that certain procedures that do not demand the use of the oscillating saw or prolonged diathermy, such as arthroscopy, minimally invasive surgery, and certain soft tissue procedures are believed to be relatively safer than those that require these types of equipment.

### Post-operative strategies:

Post-operatively, additional care should be provided such that patients are free from cross-infection. Moreover, every necessary step should be taken to discharge the patient as early as possible so that the rest of the post-operative period can be spent at home^19^.

To continue providing post-operative care,

1. Surgeons can tele-consult with patients and refrain them from visiting the hospital unless it is absolutely necessary.
2. Patients should be remotely enquired for their daily temperature and symptoms of COVID-19, if any.

**Fig. 1:**
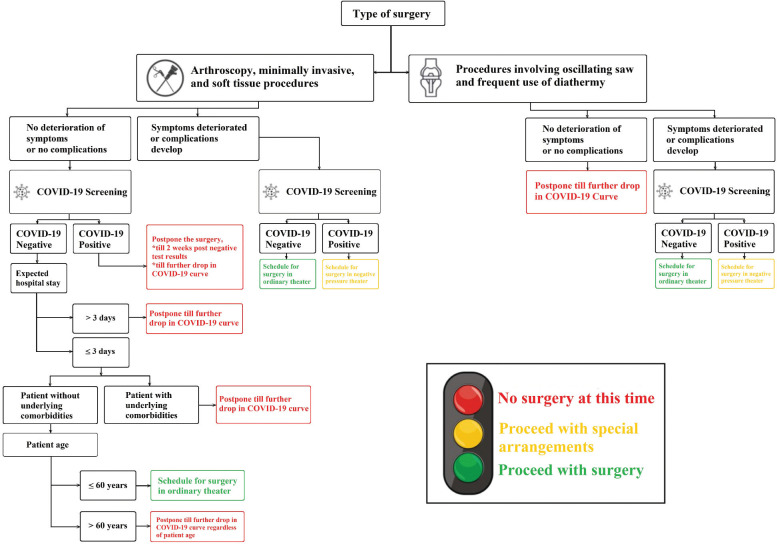
A simple traffic light system to aid in patient selection for elective orthopaedic surgery amid COVID-19 pandemic.

Fig. 1 summarises an easy to follow flowchart diagram to arrive at a decision. We have adopted a simple traffic light system to break down the possible decision into:

1. Red: No surgery at this time until further drop in the number of local COVID-19 cases
2. Yellow: Surgery may be performed if necessary, provided that additional requirements can be met
3. Green: Surgery can be done as benefits most likely outweigh the risk

## Discussion

Resuming elective orthopaedic surgery is every surgeon's intention. However, this cannot be done hastily. Given the long period of the halt in elective operations, cases have accumulated, and the waiting list is humongous^5^. While not everyone can be operated immediately after the pandemic situation eases, a sophisticated strategy is required. Available literature suggests that this strategy should not be just based on the number of pending cases but on the disease status in the community, the status of lockdown, infrastructure, healthcare manpower, and availability of various resources, including regional testing capabilities.

While non-COVID-19 patients can be operated with minimal risk, operating on a COVID-19 patient needs the utmost care to avoid potential staff morbidity. Only negative pressure theatres should be used for such patients as air from inside the theatres should not escape outside into hallways and corridors, risking those using such areas. Separate pathways to operating theatre for COVID-19 patients should be assigned. Personnel during all stages of their procedure should be kept to minimum^12,14,23^. Surgeons and assisting personnel should remain cautious while using bone drills, and oscillating saws as there is a chance for a splash of body fluids. Besides, electrocautery, either in cutting or coagulation modes, should be wisely used, as they can be a source of aerosolization^24^. Hence, most sources suggested that minimally invasive, arthroscopy and soft tissue procedures may represent the first stage of resuming electives^7,16^.

Despite effective control measures, the risk of COVID-19 transmission cannot be eradicated entirely, especially in a hospital set up that deals with COVID-19. Hence, appropriate informed consent must be obtained that clearly explains the chance of infection and also that the plan of action is tentative and subject to change depending on the local situation^18^.

Hospitals can take their decision to resume activities based on the guidelines that this review summarises, but this may not be an easy task as it requires additional human resources and finances^25^. Moreover, the focus should not just be on resuming activity but also on preparedness for a second wave^26,27^. For this reason, a stockpile of essential resources should be maintained, and manpower needs to be deployed wisely, giving special considerations to illness, fatigue, and social issues among healthcare workers.

While it is essential to follow guidelines, they are not a “one size fits all”. Occasionally, what is applicable in one setting may not be practical in another due to one or more reasons such as lack of resources, workforce shortage, etc. Hence, it is necessary to improvise own strategies with what is available. In such circumstances, surgeons should consider risk-benefit ratios and be wary of taking any risk that could jeopardise the safety of oneself and others.

## Conclusion

As COVID-19 palliates, it is instrumental that organisations have taken the lead to provide guidelines to bring the best practices to every healthcare professional. In this extraordinary time, guidelines come in as a relief for every surgeon who is in a dilemma whether to resume electives or not. Putting safety first, these guidelines or suitable versions should be followed at all levels wherever possible to avoid the lack of trained manpower in the event of staff morbidity. Every healthcare professional should be their own judge to see that they adhere to the guidelines on all occasions.
